# The growing number of female physicians: meanings, values, and outcomes

**DOI:** 10.1186/2045-4015-2-47

**Published:** 2013-12-19

**Authors:** Susan P Phillips

**Affiliations:** 1 Departments of Family Medicine and Public Health Sciences, Queen’s University, 220 Bagot St., K7L 5E9, Kingston, Canada

## Abstract

Throughout the developed world the proportion of women in professions such as medicine is increasing. This commentary uses Haklai et al’s nuanced report on the feminization of medicine in Israel as a starting point. I discuss whether gender shifts are an outcome of more egalitarian attitudes towards women overall, or instead arise from men choosing other professions, the extent of the shift, and its meaning for the quantity and quality of medical care. The discussion is embedded in more fundamental concepts such as the aims of medical practice and the best indicators of effective care. I reflect on concerns about lower female physician productivity at a time when the proportion of female physicians still remains below parity in almost all countries. Medicine is embedded in the principles and expectations of the community being served. The profession’s values and practices both shape and are shaped by those of that larger community. As cultures move toward equality, proportional representation of women and men in medicine will follow, while remaining limitations to gender equality will be mirrored in opportunities and restrictions for women in medicine. This is a commentary on http://www.ijhpr.org/content/2/1/37/.

## 

"Female physicians: trends and likely impacts on healthcare in Israel" analyzes recent demographic shifts among Israeli physicians, documenting the growing proportion of women among medical students, immigrant physicians and some specialists, although not among Israelis who study medicine abroad [1]. In an objective and balanced manner, the authors also discuss potential risks and benefits of the gender shift. Their paper is refreshingly at odds with a growing body of research and commentary that often implicitly (and sometimes explicitly) suggests that an influx of women brings a decrease in productivity, discriminates against men, and may devalue the medical profession. Using traditional male practice patterns and proportions as their norm, some earlier writing on the topic has found that relative to men, women physicians spend longer with individual patients, work fewer hours, and take more leave, particularly for parenting [2-4]. From a productivity perspective, female doctors do fall short of the male benchmark and this is a recurrent theme in the literature. Few papers consider quality of care, equality or fairness and fewer still, flip the paradigm to ask whether the demographic shift is about men opting out of medicine or women gaining access.

I wonder whether the alarm bells about an influx of women into the sciences in general and medicine in particular could be considered as moving forward looking through a rear view window, marching backwards into the future. Are we trying to fit the future to an imperfect past? Might patient health and satisfaction be more appropriate outcome measures than counting numbers of patients seen by a given doctor? There is no evidence that quantity or number of patient visits is related to quality of care delivered or to improved health. One could, in fact, hypothesize an inverse relationship between number of patients seen in a given timeframe and excellent care. Similarly, in developed countries physician density (that is, the ratio of physicians to population size) seems to have no impact on population health [5-7]. Why, then, is the baseline for comparison in much of the literature on gender shifts and trends within the medical profession a historic model of male physicians working extraordinarily long hours and spending limited time with each patient?

Relative to their male counterparts, women physicians as a group do appear to be more patient centered, communicate more effectively, and address prevention more frequently, all behaviors that seem beneficial for patients and population health [8]. Since most current practitioners, whether male or female, will have trained in a male dominated, educational milieu, it seems likely that these differences in practice patterns arise from personal and gender-related traits rather than educational imperatives.

I am cautious about dichotomizing men and women, that is, about assuming homogeneity of values and behaviors within each group and large differences between sexes. Nevertheless, it would appear that there is something about being female that makes for good doctoring and that sustains regardless of training. With optimism not grounded in evidence, I wonder whether, and hope, that as women infiltrate academic medicine their patient-centered approach to care will change practice patterns and approaches of all trainees, helping them see the individual hidden behind the homogenized common patient of clinical practice guidelines. I fear that gaining entry to the profession may instead change the women who increasingly choose to become doctors.

The gender shift described by Haklai et al. [1] and the angst such shifts have engendered elsewhere is not unique to medicine or to Israel. Throughout the developed world women are approaching parity in science related professions. Shifts in values away from discrimination and towards equality and fairness percolate through all aspects of most developed countries, including higher education and the professions. The following rhyme, taken from a McGill University (Montreal, Canada) publication (1902) now seems laughable,

“You're a girl” said the youth "and you put great reliance On Men when you're frightened by Mice. But to take up the study of Medical Science! Do you think, for a girl, it is nice?"

However, some traditional sex roles and expectations remain and will continue to shape medicine’s position within society and women’s horizons within medicine. At a societal level, there have been musings, although these generally remain unwritten as they fly in the face of egalitarian values, that an influx of women will erode medicine’s power and prestige. For individual female doctors, the realities of disproportionate responsibility for family formation and obligations still determine specialty choice, pushing them towards programs with shorter training and more flexible work schedules [1]. Stereotypic values of teachers continue to discourage women from attempting entry into traditionally male specialties. The option for women doctors of all ages to work fewer hours than men reflects positively on Israel’s valuing of family and children but may also speak to gendered expectations regarding responsibility for family and child care.

The data from Haklai et al’s study indicate that Israel is not immune to a global trade in medical education and practice. Increasingly, in developed countries those who can afford it send their children to “off shore” schools when those children are not admitted into medical school in their own countries [9]. The result is a new two-tier education system with several “two-tiers”; male – female (as Israelis studying abroad are overwhelmingly male), have – have-not (only the wealthy can pay the tuition fees charged by these for-profit medical schools), high quality local education for those students with the highest credentials versus students with less academic ability educated at schools of lower caliber in poorer countries. This globalization of medical education co-exists with an international migration of doctors from the poorest and/or most repressive of countries to less poor ones, to middle income countries, to developed countries and, finally, to the US, Canada and western Europe [10]. In Israel it appears that place of study partially determines the gender mix of doctors. If Israel suddenly stopped granting medical licenses to Israelis who studied medicine abroad, the proportion of female physicians would increase further. A skeptic might say that reporting such information has the potential to fuel an argument supporting credentialing of those who study abroad as a smokescreen for maintaining the existing predominance of men in medicine. Conversely, those who favor increasing the proportion of women in medicine may see an opportunity to do so by capping licenses to practice offered to those who study abroad, rather than entering the murky waters of the debate on the feminization of medicine.

What do Haklai et al’s analyses say about the society in which their study is embedded? The combination of an increasing proportion of women in medicine and no fall in the status of the profession suggests that gender equality is a fundamental value in Israel. However, as noted earlier, “female” is not a uniform category with respect to opportunity, and the data speak to this. Women make up only 13% of Israeli Arab physicians. They also comprise a minority of Israelis sent abroad for what is generally an expensive “back door” into medicine. Overall, the proportion of women in medicine has risen from 38 to 42 percent in 12 years. A small absolute change, fed by, although not limited to immigration from Russia, one that still falls short of parity, and that may have reached a plateau.

Change often involves a tug of war between two paradigms. Equalizing opportunities for women tugs societies toward egalitarianism while at the same time old stereotypes that devalue women’s work push back against this. Which will prevail remains to be seen – will women’s increasing presence in medicine add to social equality overall or will it become a reason for the devaluing of medicine as a profession? I believe that behind the research on shifting demographics in medicine lies an even more fundamental question and needed change. Using quantities such as physician density and numbers of patient encounters as outcome indicators of quality misses the measure of what medicine is all about, which to my way of thinking is improving health of individuals and populations rather than merely demonstrating access to care.

## Competing interests

The author has no competing interests to declare.

## Authors’ information

Susan P Phillips is a family physician and Professor in the School of Medicine at Queen’s University, Kingston, Canada. Her research focus is gender as a determinant of health and the roles gender plays in medical education and practice.

## Commentary on

Haklai Z, Applbaum Y, Tal O, et al. Female physicians: trends and likely impacts on healthcare in Israel. Isr J Health Policy Res 2013, 2:37XX
